# The efficacy of multiple versus single hyaluronic acid injections: a systematic review and meta-analysis

**DOI:** 10.1186/s12891-017-1897-2

**Published:** 2017-12-21

**Authors:** Andrew Concoff, Parag Sancheti, Faizan Niazi, Peter Shaw, Jeffrey Rosen

**Affiliations:** 1 Departments of Orthopedics and Rheumatology, St. Jude Medical Center, Fullerton, California, USA; 2 Sancheti Institute for Orthopaedics and Rehabilitation, Maharashtra, India; 3 Ferring Pharmaceuticals Inc., Parsippany, NJ USA; 4 Department of Orthopaedics & Rehabilitation, New York Presbyterian Queens; Department of Clinical Orthopaedic Surgery, Weill Medical College of Cornell University, New York, NY USA

**Keywords:** Intra-articular hyaluronic acid (IA-HA), Osteoarthritis (OA), Knee

## Abstract

**Background:**

Intra-articular hyaluronic acid (IA-HA) is a common therapy used to treat knee pain and suppress knee inflammation in knee osteoarthritis (OA), typically prescribed in regimens ranging from a single injection to 5 weekly injections given once weekly. We conducted a systematic review to determine the efficacy of IA-HA, with subgroup analyses to explore the differences in knee pain and adverse events (AEs) across different dosing regimens.

**Methods:**

We conducted a systematic search of the literature to identify studies evaluating IA-HA for the management of knee OA compared to IA-saline. Primary outcome measure was the mean knee pain score at 13 Weeks (3 months) or 26 weeks (6 months). Secondary outcome was the number of treatment-related AEs and treatment-related serious adverse events (SAEs). We evaluated differences in levels of pain and AEs/SAEs between dosing regimens compared to IA-Saline.

**Results:**

Thirty articles were included. Overall, IA-HA injections were associated with less knee pain compared to IA-Saline injections for all dosing regimens. 2–4 injections of IA-HA vs. IA-Saline produced the largest effect size at both 3-months and 6-months (Standard mean difference [SMD] = −0.76; −0.98 to −0.53, 95% CI, *P* < 0.00001, and SMD = −0.36; −0.63 to −0.09 95% CI, *P* = 0.008, respectively). Additionally, single injection studies yielded a non-significant treatment effect at 3 and 6 months, while ≥5 5 injections demonstrated a significant improvement in pain only at 6 months. Five or more injections of IA-HA were associated with a higher risk of treatment-related AEs compared to IA-Saline (Risk ratio [RR] = 1.67; 1.09 to 2.56 95% CI, *p* = 0.02), which was a result not seen within the 1 and 2–4 injection subgroups.

**Conclusion:**

Overall, 2–4 and ≥5 injection regimens provided pain relief over IA-Saline, while single injection did not. Intra-articular injections of HA used in a 2–4 injection treatment regimen provided the greatest benefit when compared to IA-Saline with respect to pain improvement in patients with knee OA, and was generally deemed safe with few to no treatment-related AEs reported across studies. Future research is needed to directly compare these treatment regimens.

**Electronic supplementary material:**

The online version of this article (10.1186/s12891-017-1897-2) contains supplementary material, which is available to authorized users.

## Background

Knee osteoarthritis (OA) is a slowly progressive joint disorder characterized by joint pain, cartilage degeneration, and inflammation that affects approximately 250 million people worldwide [1]. Knee OA leads to negative impacts on socioeconomic factors including impaired work performance and early retirement [2, 3].

Multiple treatment options are available for knee OA, ranging from conservative management to total knee arthroplasty. Common non-operative therapies include non-steroidal anti-inflammatory drugs (NSAIDs), physical therapy, analgesics such as acetaminophen, and intra-articular (IA) therapies such as corticosteroids and hyaluronic acid (HA) [4]. IA-HA products have been used in the United States as a treatment for knee OA since 1997 and are approved by the Food and Drug Administration (FDA) [5]. The putative mechanisms of action through which IA-HA provides therapeutic effects include anti-inflammatory effects, chondroprotection, proteoglycan synthesis, and shock absorption properties [6]. IA-HA is frequently used in clinical practice to treat knee pain and suppress knee inflammation.

In 2013, the American Academy of Orthopaedic Surgeons (AAOS) recommended against the use of IA-HA for knee OA [7]. However, more recently, certain physician-specialty societies have recommended the use of IA-HA in treating knee OA. The American College of Rheumatology (ACR) recommended IA-HA for knee OA patients who had an inadequate response to initial therapy in a recent position statement [8]. The American Medical Society for Sports Medicine (AMSSM) recommended the use of HA for the appropriate patients with knee OA based on recent evidence in a network meta-analysis [9]. The European Society for Clinical and Economic Aspects of Osteoarthritis (ESCEO) task force issued a consensus statement recommending the use of IA-HA in knee OA patients with mild to moderate disease, and for more severe patients who are not good candidates for total knee replacement surgery or wishing to delay the surgical procedure [10]. The OA Research Society International (OARSI) has an uncertain recommendation for the use of IA-HA in treating knee OA based on the good level of quality of evidence available, suggesting physicians discuss the risk-benefit profile of IA-HA along with individual characteristics, comorbidities and preferences of the patient [11]. Despite the clinical considerations and availability of evidence recommending the use of IA-HA in treating knee OA, the optimal treatment regimen and patient selection criteria have yet to be determined.

IA-HA is commonly prescribed in different injection regimens, which vary from a single injection to one injection a week for 5 weeks. Multiple randomized controlled trials (RCTs) have been conducted evaluating different dosing regimens of IA-HA versus IA-Saline. We conducted a systematic review of the published literature to determine the efficacy of IA-HA vs IA-Saline in patients with knee OA, with subgroup analyses to explore the differences in levels of pain, adverse events (AEs), and serious adverse events (SAEs) across different dosing regimens.

## Methods

### Literature search

A comprehensive literature search for relevant articles was conducted on February 26th, 2016 using a detailed search of the MEDLINE and EMBASE and PubMed databases (Additional file 1). The Preferred Reporting Items for Systematic Reviews and Meta-Analyses (PRISMA) 2009 checklist was applied a posteriori to ensure appropriate reporting of methods and results [12]. The inclusion criteria were: 1) blinded randomized controlled trial (RCT) comparing IA-HA with intra-articular saline (IA-Saline) injection; 2) knee pain was a reported outcome; and 3) articles that were published in English. Title screening, abstract screening and full text screening were conducted in duplicate.

### Data abstraction

We abstracted details on the study characteristics, details about the HA product used (manufacturer, production method (Bio-HA [biologically derived/non– animal stabilized] or AD [avian-derived]) and molecular weight (indicated as high if ≥3000 kDa, moderate if <3000 and ≥1500 kDa, or low if <1500 kDa), the timing of injections, reported pain outcomes, and safety data (the number of treatment-related AEs and treatment-related SAEs). Data extraction was completed by one reviewer, and another reviewer completed a review of the data for accuracy.

### Outcome measures

The primary outcome measure was the mean knee pain score at the reported follow-up nearest to 13 Weeks (3 months) or 26 weeks (6 months). The Western Ontario and McMaster Universities Arthritis Index (WOMAC) Pain scores were extracted whenever reported. If WOMAC pain scores were not reported an a priori hierarchy of outcomes was used to extract the next-most relevant outcome measure. The hierarchy used was taken from a previous meta-analysis, and is as follows: WOMAC Pain, Visual Analog Scale (VAS) Pain with activity/walking, VAS Pain weight bearing, VAS pain at rest, Other Pain outcomes (Knee Injury and Osteoarthritis Outcome Score (KOOS), Musculoskeletal Outcomes Data Evaluation and Management System (MODEMS), Index of Severity for Osteoarthritis for the Knee (ISK) assessment), WOMAC Total Score [13].

We extracted safety data from each trial on total number of participants experiencing treatment-related adverse events (AEs) and total number of treatment-related serious adverse events (SAEs). If data for these safety measures was not reported, the corresponding study was not included in the meta-analysis for that specific safety measure. Data from the intent-to-treat population was used whenever possible. Data extraction was completed in duplicate by two reviewers.

### Data analysis

The bias corrected (Hedges) effect size for each trial was calculated using an online Excel calculation tool [14]. If the article did not provide the mean pain score and standard deviation/standard error, then the effect sizes were obtained from recently published systematic reviews and meta-analyses [4, 15]. Standard error was calculated from the confidence intervals (CI) of the effect sizes taken from the meta-analysis using the following equation:

$$ Standard error=\frac{High\ C.I. value- Low\ C.I. value}{3.92} $$

Effect size results and AEs were analyzed using the Cochrane Review Manager 5.3 software [16]. Negative effect size estimates represent benefit of IA-HA, while positive effect size estimate values represent benefit of IA-Saline. Effect size and AE analyses were separated and pooled based on the number of injections of IA-HA and IA-Saline (single injection, 2–4 injections, or ≥5 injections). Effect size estimates were analyzed using a generic inverse variance statistical method and a random effects analysis model with a 95% confidence interval for study and total effect size. Effect sizes were reported as a standard mean difference outcome measurement. The number of participants experiencing a treatment-related adverse event and treatment-related SAEs were analyzed under a dichotomous outcome assessment using Mantel-Haenzel statistical method and a fixed effects analysis model with a 95% confidence interval for study and total effect size. Heterogeneity within the included trials was measured using the I^2^ statistical measurement. Two reviewers independently graded the methodological quality of each included study using the Cochrane Collaboration’s Risk of Bias tool. The Cochrane Risk of Bias tool separates judgments about risk of bias from inadequate reporting of methodology. *Post-hoc* funnel plot analyses at 3-months and at 6-months were conducted to assess publication bias (Additional file 2: Figure S1 and Additional file 3: Figure S2).

A *post-hoc* subgroup analysis was conducted comparing the efficacy of IA-HA by total dose administered to determine whether repeated injection or total dose received likely explained the differences observed by number of injections. Studies were separated based on the total dose of IA-HA participants were given (0–60 mg, 61–100 mg, >100 mg).

### Sensitivity analysis

A sensitivity analysis was conducted to determine if single-blinded studies had a significant impact on the total treatment effect of IA-HA on knee-pain vs IA-Saline. To accomplish this, single-blinded studies were removed from analyses to determine if they had a significant impact on treatment efficacy. Another sensitivity analysis was conducted to determine if removing each study from the 3-month and 6-month treatment effect meta-analyses converted a statistically significant combined difference into a nonsignificant difference.

## Results

### Search strategy

**Fig. 1:**
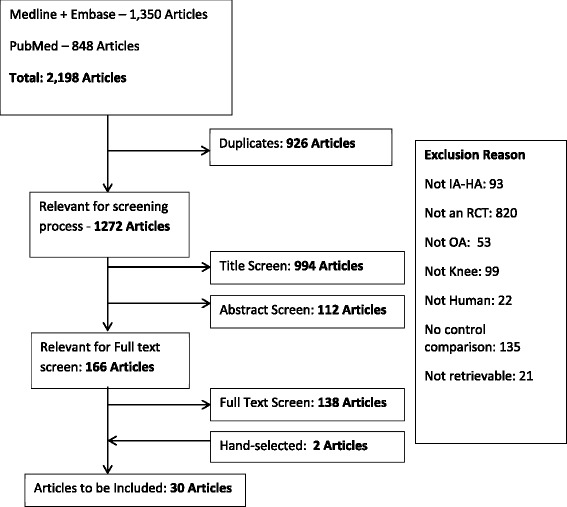
Screening process. Legend: IA-HA: Intra-articular hyaluronic acid, RCT: Randomized controlled trial, OA: Osteoarthritis

Our literature search identified 2198 articles and 166 of these articles were deemed relevant following the title review (Fig. 1). Of these, 28 articles met the pre-defined inclusion criteria. One study used arthrocentesis as a comparator and was included after careful review [17]. Two additional articles were identified from our review of the reference lists of relevant articles. Therefore, 30 articles were included in our systematic review [17–46].

### Study characteristics and demographics

Most studies were published within the last decade, and were most frequently conducted in Europe (63.3%), followed by North America (23.3%), Asia (10.0%) and Australia (3.3%) (Table 1). There were 26 double-blinded RCTs (86.7%) and four single-blinded RCTs (13.3%) included in this review (Table 2). A total of 5848 patients are included in our analysis. Four studies (*N* = 1196) used single injections of IA-HA, 16 studies (*N* = 2865) used 2–4 injections, and 11 studies (*N* = 1847) evaluated ≥5 injections. One study (*N* = 63) reported administering 1–11 injections of IA-HA and was included in both the 2–4 injections subgroup and ≥5 injections subgroup [27]. The most common follow-up period was 26 weeks (6 months) (ranging from 4 weeks to 52 weeks).

**Table 1:** Study Location and Year of Publication

Characteristic	Total (%) (*N* = 30)
Year of Publication	
1985–1989	1 (3.3)
1990–1994	4 (13.3)
1995–1999	3 (10.0)
2000–2004	6 (20.0)
2005–2009	8 (26.7)
2010–2014	7 (23.3)
2015–2016	1 (3.3)
Study Location	
Europe	19 (63.3)
North America	7 (23.3)
Asia	3 (10.0)
Australia	1 (3.3)
South America	0 (0.0)
Africa	0 (0.0)

**Table 2:** Study characteristics

Study Characteristics									
Trial ID	Country	Study Design	Funded By Industry	N	Treatment Arms		Number of injections	OA Severity (KL)	Visit closest to 26 weeks (weeks)
					Treatment	Control			
Altman, 2004	USA, Canada, Sweden	Double-blinded RCT	Yes	346	Durolane	Saline	1	2,3,4	26
Altman, 2009	USA	Double-blinded RCT	Yes	588	Euflexxa	Saline	3	2,3	26
Arden, 2014	Sweden, German, UK	Double-blinded RCT	Yes	218	Durolane	Saline	1	2,3	6
Brandt, 2001	USA	Double-blinded RCT	Yes	226	Orthovisc	Saline	3	NR	27
Chevalier, 2010	UK, France, Czech Republic, Germany, Belgium, Netherlands	Double-blinded RCT	Yes	253	Synvisc	Saline	1	2,3,4	26
Creamer, 1994	UK	Single-blinded RCT	Yes	12	Hyalgan	Saline	5	2,3,4	5
Day, 2004	Australia	Double-blinded RCT	Yes	240	Artzal	Saline	5	NR	18
DeCaria, 2012	Canada	Double-blinded RCT	Yes	30	Low MW HA	Sham HA	3	NR	26
Diracoglu, 2009	Turkey	Double-blinded RCT	No	63	Synvisc	Saline	3	2,3	4
Dixon, 1988	UK	Double-blinded RCT	Yes	63	Hyalgan	1/100 Hyalgan	1–11	NR	25
Henderson, 1994	UK	Double-blinded RCT	No	91	Hyalgan	Saline	5	2,3,4	5
Huang, 2011	Taiwan	Double-blinded RCT	Yes	200	Hyalgan	Saline	5	2,3	25
Huskisson, 1999	UK	Single-blinded RCT	No	100	Hyalgan	Saline	5	2,3	26
Jorgensen, 2010	Denmark	Double-blinded RCT	No	337	Hyalgan	Saline	5	NR	26
Karlsson, 2002	Sweden	Double-blinded RCT	Yes	246	Synvisc	Saline	3	NR	26
Kotevoglu, 2006	Turkey	Double-blinded RCT	No	78	Synvisc	Saline	3	2,3,4	27
Lohmander, 1996	Denmark, Finland, Norway, Sweden	Double-blinded RCT	Yes	240	Artzal	Saline	5	NR	20
Lundsgaard, 2008	UK, Netherlands	Double-blinded RCT	No	308	Hyalgan	Saline	4	1,2,3,4	26
Navarro-Sarabia 2011	Spain	Double-blinded RCT	Yes	306	Adant	Saline	5	2,3	28
Neustadt, 2005	USA, Canada	Double-blinded RCT	Yes	229	Orthovisc	Arthrocentesis	4	1,2,3	22
Petrella, 2006	Canada	Double-blinded RCT	Yes	106	HA sodium salt	Saline	3	1,2,3	12
Petrella, 2008	Canada	Double-blinded RCT	No	200	Synvisc	Saline	3	1,2	16
Pham, 2004	France	Double-blinded RCT	No	301	NRD101 HA	Saline	3	1,2,3,4	52
Puhl, 1993	Germany	Double-blinded RCT	Yes	209	Artzal	1/100 ARTZ	5	NR	14
Scale, 1994	USA	Double-blinded RCT	Yes	80	Synvisc	Saline	2, 3	2,3,4	12
Sezgin, 2005	Turkey	Single-blinded RCT	No	41	Orthovisc	Saline	3	2,3	4
Strand, 2012	Japan	Double-blinded RCT	Yes	379	Gel-ONE	Saline	1	1,2,3	13
Tamir, 2001	Israel	Single-blinded RCT	No	49	BioHY	Saline	5	2,3,4	20
van der Weegen, 2015	Netherlands	Double-blinded RCT	Yes	196	Fermathron plus	Saline	3	1,2,3	26
Wobig, 1998	Germany	Double-blinded RCT	Yes	110	Synvisc	Saline	3	1,2,3	26

Low molecular weight HA was used most frequently (47%), followed by high molecular weight HA (43%) and moderate weight HA (10%) (Table 3). The majority of IA-HA products (63.3%) were produced via avian-derived molecules (ADHA), and through bacterial processes of biological fermentation (Bio-HA) (33.3%). One study (3.3%) did not report the IA-HA product used. More than half of the included studies did not report the injection method used (64.5%); however, the most reported method of injection was either a lateral or medial approach. Injection regimens were fairly consistent amongst treatment groups, reporting similar concentrations of HA preparations (approximately 10 mg/2 ml to 30 mg/3 ml) and volume of HA and saline solutions administered (2.0 ml to 3.0 ml range).

**Table 3:** Treatment characteristics

Trial ID	Molecular Weight	Cross-Linked	Production method	Injection technique	Injection regimen
Altman, 2004	High	Yes	Bio-HA	NR	Single 60 mg/3 ml injection of IA-HA or saline
Altman, 2009	High	No	Bio-HA	Suprapatellar or infrapatellar approach	3 weekly injections of IA-HA (20 mg/2 ml) or saline
Arden, 2014	High	Yes	Bio-HA	Lateral midpatellar, lateral upper-patellar or medial	Single 3 ml injectionof IA-HA (60 mg/3 ml) or saline
Brandt, 2001	Moderate	No	Bio-HA	NR	3 weekly injections of IA-HA (2 ml; 15 mg/ml) or saline (2 ml)
Chevalier, 2010	High	Yes	ADHA	NR	Single 6 ml injectionof IA-HA or saline
Creamer, 1994	Low	No	ADHA	NR	Single injection of HA (20 mg/2 ml) into one knee and saline (2 ml) into the other
Day, 2004	Low	No	ADHA	Lateral or medial approach	5 weekly injections of 25 mg/2.5 ml IA-HA in a phosphate buffered solution or 2.5 ml vehicle or saline
DeCaria, 2012	Low	No	NR	Anteromedial approach	3 weekly injections of IA-HA (2 ml of 20 mg/ml), or saline (1.2 ml of 0.001 mg/ml HA)
Diracoglu, 2009	High	Yes	ADHA	NR	3 weekly injections of IA-HA or saline
Dixon, 1988	Low	No	ADHA	NR	Up to 11 injections of IA-HA (20 mg/2 ml) or placebo (0.2 mg sodium hyaluronate)
Henderson, 1994	Low	No	ADHA	Medial approach	5 weekly injections of IA-HA (20 mg/2 ml) or saline
Huang, 2011	Low	No	ADHA	NR	5 weekly injections of IA-HA (20 mg/2 ml) or saline
Huskisson, 1999	Low	No	ADHA	NR	5 weekly injections of IA-HA (20 mg/2 ml) or saline
Jorgensen, 2010	Low	No	ADHA	Lateral parapatellar approach	5 weekly injections of IA-HA (20 mg/2 ml) or saline
Karlsson, 2002	High	Yes	ADHA	NR	3 weekly injections of IA-HA (Artzal, 2.5 ml or Synvisc, 2.0 ml) or saline
Kotevoglu, 2006	High	Yes	ADHA	Anterolateral approach	3 weekly injections of IA-HA or saline
Lohmander, 1996	Low	No	ADHA	NR	5 weekly injections of IA-HA (25 mg/2.5 ml) or saline
Lundsgaard, 2008	Low	No	ADHA	Lateral midpatellar portal	4 weekly intra-articular injections of IA-HA 2 mL (Hyalgan, 10.3 mg/ml) versus physiological saline 20 ml (distention) versus physiological saline 2 ml
Navarro-Sarabia 2011	Low	No	Bio-HA	NR	4 treatment cycles of 5 weekly injections of IA-HA or saline (2.5 ml)
Neustadt, 2005	Moderate	No	Bio-HA	Lateral or medial approach	4 weekly injections of IA-HA (30 mg/2 ml), or 4 arthrocenteses without injection (control)
Petrella, 2006	Low	no	ADHA	NR	3 weekly injections of IA-HA (20 mg/ml) or saline
Petrella, 2008	High	No	ADHA	Medial approach	3 weekly injections of IA-HA or saline
Pham, 2004	NR	NR	NR	NR	3 weekly injections of IA-HA or saline
Puhl, 1993	High	No	ADHA	NR	5 weekly injections of IA-HA (25 mg/2.5 ml) or dilute IA-HA control(0.25 mg/2.5 ml HA)
Scale, 1994	High	No	ADHA	NR	Two intra-articular injections of IA-HA (2.0 ml) administered 2 weeks apart were compared with three intra-articular injections of IA-HA given 1 week apart vs saline (2.0 ml)
Sezgin, 2005	Moderate	No	Bio-HA	NR	3 weekly injections of IA-HA (15 mg/ml) or saline
Strand, 2012	High	Yes	ADHA	NR	Single 3 ml injection of IA-HA (30 mg/3.0 ml) or saline
Tamir, 2001	High	No	Bio-HA	NR	5 weekly injections of 20 mg IA-HA (10 mg/ml) or saline
van der Weegen, 2015	Low	Yes	Bio-HA	NR	3 weekly injections of IA-HA (15 mg/ml) or saline
Wobig, 1998	High	Yes	ADHA	NR	3 weekly injections of 2.0 ml IA-HA or saline

*ADHA* Avian-derived hyaluronic acid*, Bio-HA* Biologically fermented hyaluronic acid*, IA-HA* Intra-articular hyaluronic acid*, NA* Not Applicable*, NR* Not Reported

### IA-HA versus IA-saline: Follow-up closest to 3 months (13 weeks)

**Fig. 2:**
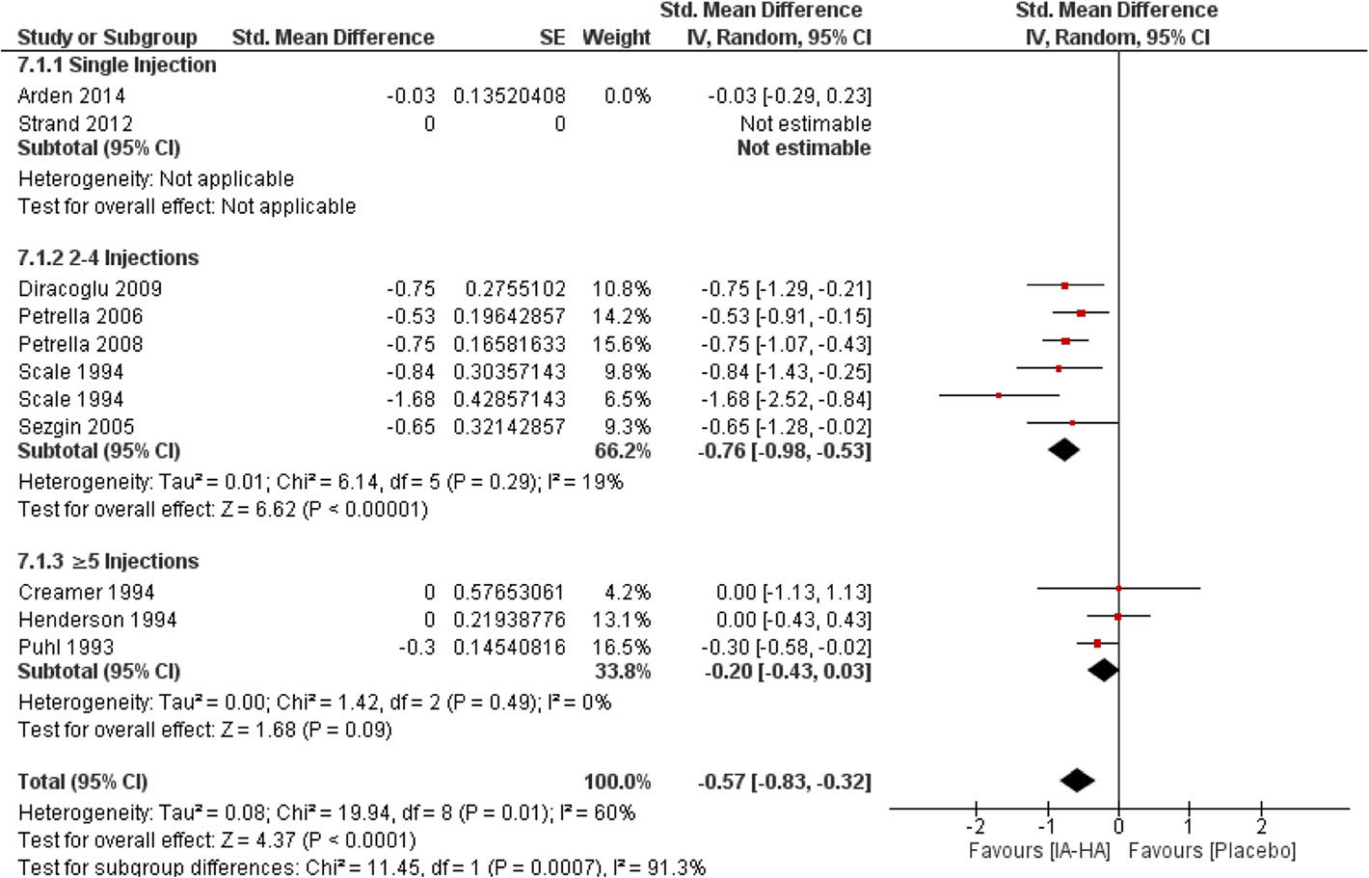
Efficacy of IA-HA injections closest to 3-months

Length of follow-up for included studies with nearest to 3-month follow up data ranged from four weeks up to 16 weeks (Fig. 2). Single injection was comprised of only one estimable study [20] (Standard mean difference [SMD] = −0.03; −0.29 to 0.23). 2–4 injections of IA-HA vs. IA-Saline produced the largest effect size of the subgroups (SMD = −0.76; −0.98 to −0.53, 95% CI, *P* < 0.00001). ≥5 injections of IA-HA vs. IA-Saline produced a non-significant effect size estimate of −0.20 (−0.43 to 0.03, 95% CI, *P* = 0.09). Test for subgroup differences were significant (P < 0.00001). Heterogeneity was only observed for studies in the 2–4 injections subgroup (I^2^ = 19%).

### IA-HA versus IA-saline: Follow-up closest to 6 months (26 weeks)

**Fig. 3:**
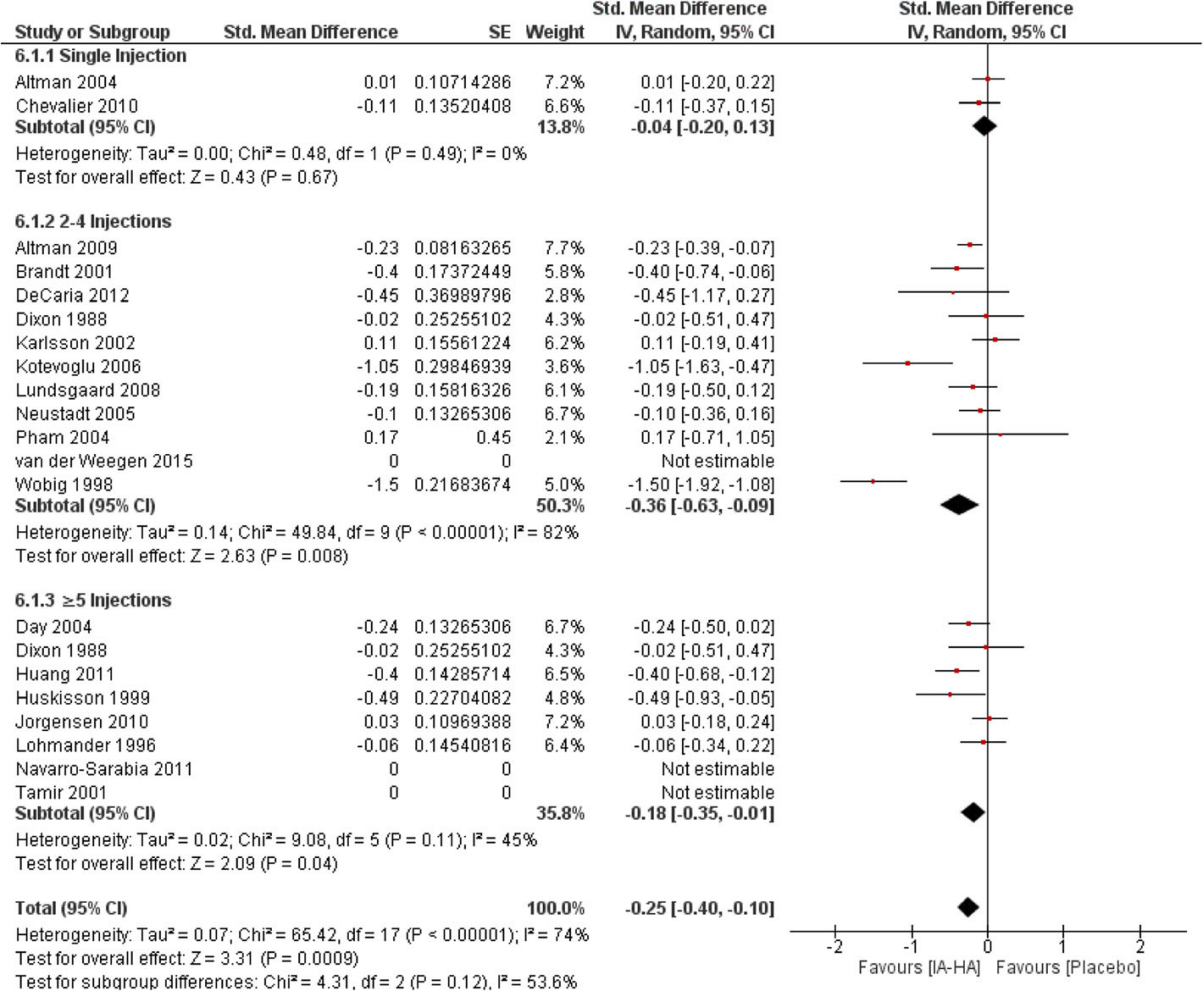
Efficacy of IA-HA injections closest to 6-months

Length of follow-up for included studies with nearest to 6-month follow up data ranged from 18 weeks up to 52 weeks (Fig. 3). Single injection studies yielded a non-significant treatment effect (SMD = −0.04; −0.20 to 0.13, 95% CI, *P* = 0.67). 2–4 injections of IA-HA vs. IA-Saline produced the largest significant effect size (SMD = −0.36; −0.63 to −0.09 95% CI, P = <0.00001). Studies with ≥5 injections of IA-HA vs. IA-Saline produced a significant effect size estimate of −0.18 (−0.35 to 0.01, 95% CI, *P* = 0.04). Heterogeneity was observed for studies in the 2–4 injections subgroup (I^2^ = 82%) and ≥5 injections subgroup (I^2^ = 74%).

### Efficacy of IA-HA vs IA-saline – Dosage comparison

**Fig. 4:**
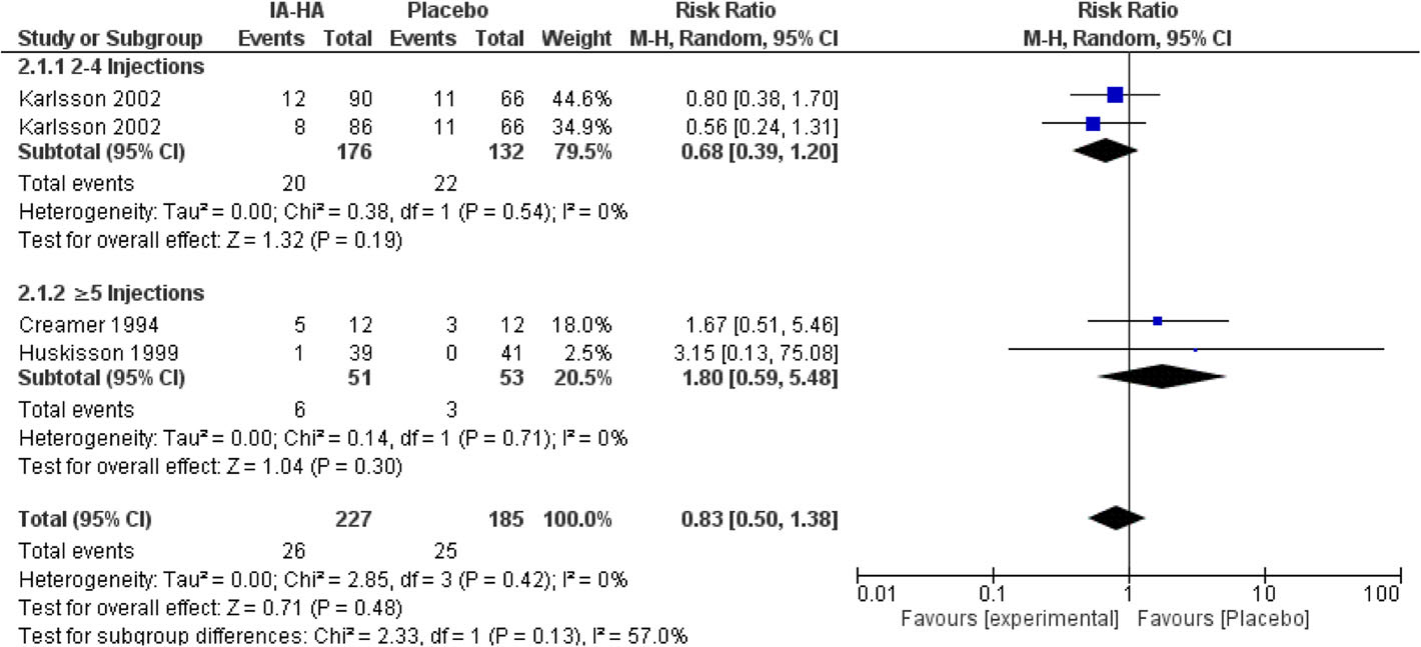
Total number of participants experiencing a treatment-related serious adverse event

No significant subgroup difference were observed when studies were analyzed by total dose of IA-HA administered (*P* = 0.90; Fig. 4). Studies failing to report dosage of IA-HA administered were removed from analysis [21, 24, 32, 33, 35, 38, 39, 46].

### Treatment-related adverse events and serious adverse events for IA-HA vs. IA-saline

**Fig. 5:**
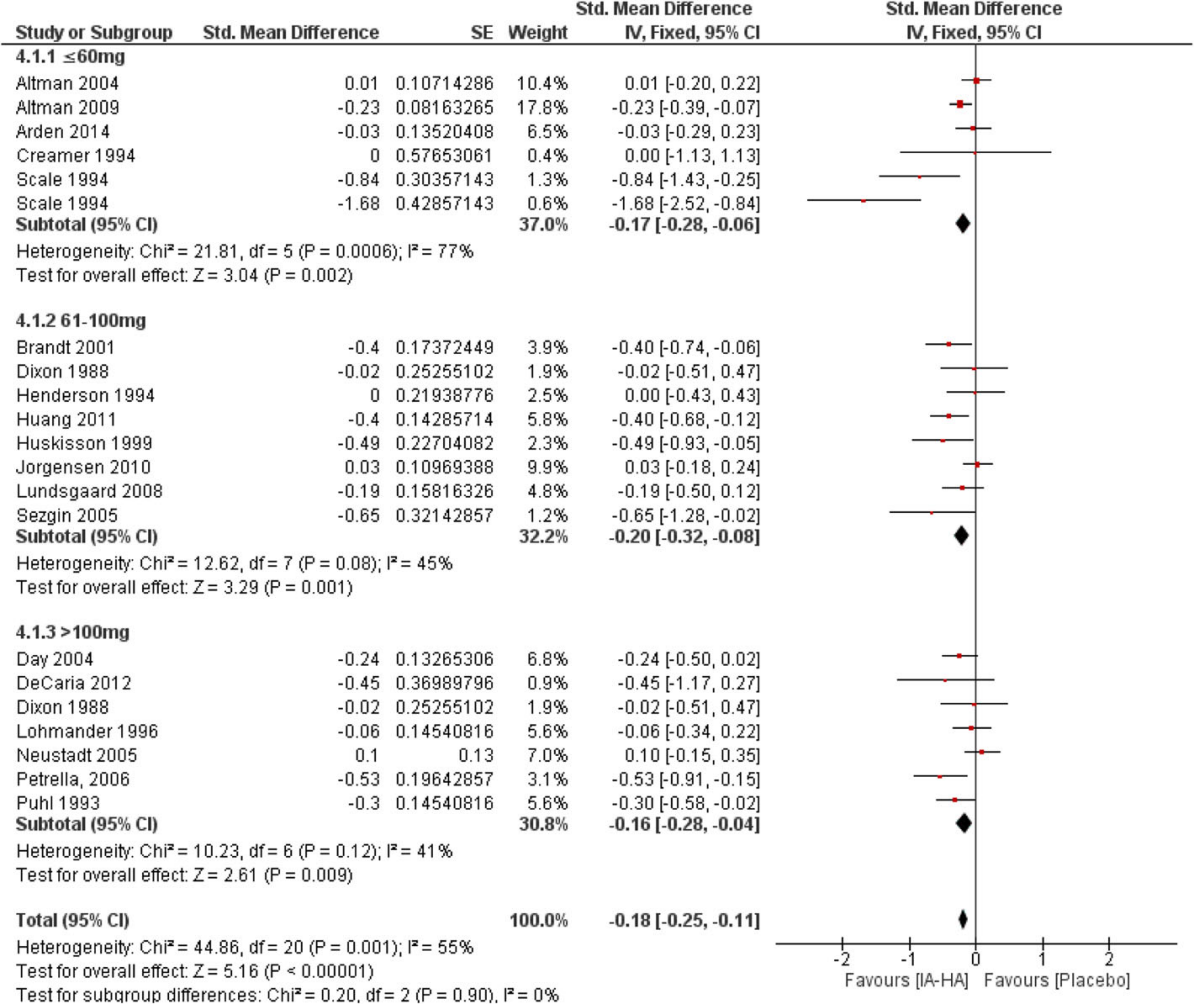
Subgroup analysis of the efficacy of IA-HA by total dose administered

**Fig. 6:**
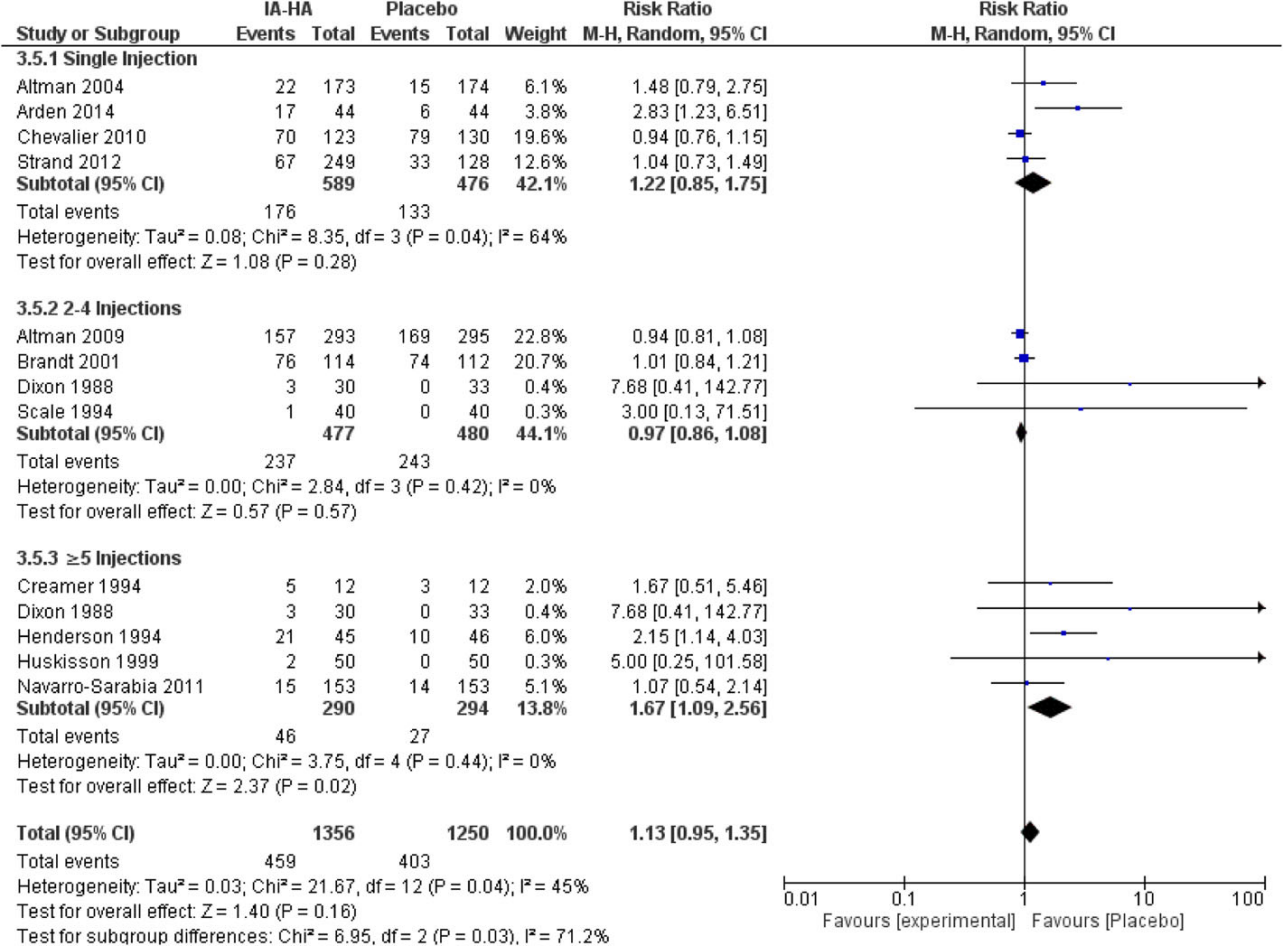
Total number of participants experiencing a treatment-related adverse event

There were no statistically significant differences in the total number of treatment-related AEs compared to saline injection for single injection of IA-HA vs IA-Saline (Risk ratio [RR] = 1.11; 0.93 to 1.32 95% CI, *p* = 0.26) or 2–4 injections of IA-HA vs IA-Saline (RR = 0.98; 0.87 to 1.09 95% CI, *p* = 0.67; Fig. 5). Studies with ≥5 injections of IA-HA had statistically more treatment-related AEs compared to IA-Saline (RR = 1.70; 1.12 to 2.59 95% CI, *p* = 0.01). Significant subgroup differences were observed between number of injections and treatment-related AEs (*P* = 0.03), but not for treatment-related SAEs (Fig. 6).

### Sensitivity analysis

Four single-blinded studies were removed from the sensitivity analysis [23, 30, 42, 44]. The pooled effect size remained statistically significant with little change in total effect size when these single-blind studies were removed from the analysis (SMD = −0.19 [−0.25, −0.13], *P* < 0.001). In studies with a follow-up closest to 6 months (26 weeks), removing one study in the ≥5 injections subgroup [24, 29, 30] changed the subgroup results from significant to non-significant.

### Risk of bias

**Fig. 7:**
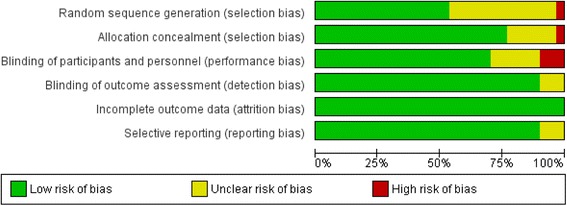
Risk of bias summary

Included studies demonstrated minimal bias with respect to categories of selection bias, detection bias, performance bias, attrition bias and reporting bias (Fig. 7). Few studies failed to report methods of randomization and methods of blinding.

## Discussion

Overall, treatment with IA-HA was observed to be more effective in treating patients with OA knee pain compared to IA-Saline, with 2–4 injections demonstrating the largest treatment effect at both 3-month and 6-month follow-ups. Single injections of IA-HA were not significantly more effective than saline at 3-month or 6-month follow ups; however there were only four published RCTs comparing single injections with one trial not reporting the effect size. The ≥5 injections subgroup demonstrated significant improvement in pain at 6 months only. These results indicate that only the 2–4 injection regimen group provided consistently significant pain relief at both 3 and 6 months follow-up, when compared to IA-Saline.

Our results showed greatest improvement at 3 months, while improvement was seen to a lesser extent at 6 months following IA-HA. This is similar to the therapeutic trajectory of IA-HA vs IA-Saline conducted by Bannuru and colleagues [47]. Effect sizes favored IA-HA by week 4 ((0.31; 95% CI 0.17, 0.45) and peak at week 8 (0.46; 0.28, 0.65), suggesting that the optimal improvement is seen around 2 months after IA-HA injection. A network meta-analysis conducted by Bannuru et al [4] found similar results when comparing IA-HA to saline controls; IA-HA was favoured over IA-Saline control (SMD = 0.429; C.I. 0.261 to 0.598, *p* = 0.000) on knee pain at 4 to 13 weeks of treatment. Another network meta-analysis demonstrated similar conclusions, as IA-HA was seen to have a significant effect on pain when compared to IA-Saline [48]. Our analysis has demonstrated that not only does the overall pooled estimate for HA as a class demonstrate a benefit for pain relief, there seem to be differences in effect as a result of the injection regimen provided. Previous analyses have suggested that in addition to HA class benefits in pain relief, molecular weight may also be a product characteristic that affects the potential outcomes of patients [13, 15].

The results of this meta-analysis of knee pain efficacy for 1 injection, 2–4 injections and ≥5 injections of IA-HA concur with a recently published RCT [49] in suggesting that the dosing regimen of IA-HA should be considered for development of future guidelines in treating symptomatic knee OA. Zoboli et al. published a head-to-head comparison, RCT assessing whether a single 6 ml application of HA has the same effectiveness as the three-weekly 2 ml dose [49]. Although this study showed no significant differences in efficacy between one single injection and three weekly injections of the same dose of IA-HA administered, the 3 weekly injections regimen showed statistically significant improvement from baseline pain (WOMAC pain and VAS) whereas the single injection regimen did not. Although there were subgroup differences between the numbers of injections administered within this meta-analysis, there was no subgroup difference in efficacy observed when comparing total dose of IA-HA administered to participants vs IA-Saline.

Recent evidence suggests products with an average molecular weight ≥ 3000 kDa provide favorable efficacy results when compared with products of an average molecular weight < 3000 kDa, and significantly fewer discontinuations compared with products with a molecular weight < 1500 kDa^13^. In this meta-analysis, we identified that the number of injections may also play a significant role in efficacy outcomes. Although the 2–4 injection subgroup included products within a wide range of MW’s the effect size was still significant compared to the single injection subgroup which consisted of only cross-linked high molecular weight (HMW) products. It has been suggested oxygen-derived free radicals act as a mediator in the inflammatory response, and that these radical species are responsible for increased HA degradation [50]. HMW products may achieve better efficacy due to an increased residency time of within the synovial fluid, producing a prolonged anti-inflammatory response within the joint, blocking inflammatory receptors, and a longer lasting chondroprotective effect (inhibition of metalloproteases, nitric oxide, and stimulation of proteoglycan/glycosaminoglycan synthesis) [51–53]. Therefore, repeated exposures of HA may perpetuate improvement in the synovial fluid environment allowing subsequent IA-HA shots to provided extended effects. The results of this study help demonstrate that, while molecular weight is an important factor in the efficacy of HA products, the number of injections provided also plays a major role in optimizing the efficacy seen within knee OA patients. Thus is particularly clear given that, although all single injection HA products were of a high MW, they did not demonstrate a reduction in pain comparable to the 2–4 injections subgroup, which included several studies of LMW HA products. This study demonstrates that receiving the typical 3 injection regimen of a HA may be more effective than a single injection high molecular weight product. Future aims should investigate the mechanism of actions of high molecular weight HA in multiple injections compared to a single injection.

Significantly more treatment-related AEs were observed in participants receiving ≥5 injections vs IA-Saline; a result not seen in the single and 2–4 injection categories. Although this comparison to IA-Saline was significant, the subgroup analysis comparing the different injection regimens did not show a significant difference in treatment-related AEs between numbers of injection subgroups. Moreover, four of the five studies that reported treatment-related AEs reported a difference of only 1–3 events between treatment groups, and this analysis was largely subjugated by a single study conducted by Henderson et al.^256^ that reported a difference of 11 events, with the IA-HA treatment group experiencing more AEs. This meta-analysis observed very few serious treatment-related AEs, with little to no events reported for all injection regimens. Our findings are similar to the meta-analysis conducted by Miller and Block [54] that focused on the safety and efficacy of US approved IA-HA products in saline-controlled trials, where no SAEs were determined related to injection of HA or saline.

This review has strength in its methodological approach in systematically identifying available saline-controlled RCTs from online databases. The use of a thorough and systematic approach to article selection and data abstraction provides further strength to this report. A search of the grey literature or unpublished literature was not conducted; however, authors scanned references in articles that met the inclusion criteria for literature not captured in the search. Limitations of this review include that some studies of IA-HA treatment were excluded from the analysis due to not reporting efficacy measurements for knee pain. Another limitation is the lack of a direct comparison between the numbers of injections received, as the literature is not robust enough to permit such analysis. Additionally, the majority of these RCTs were industry funded with a moderate to high risk of bias. Further, the inconsistent reporting of pain scores, along with the variable length of follow-up time between studies provides another limitation with respect to our pooled results. Heterogeneity was also seen within some subgroups, which is an additional limitation to this study. Finally, no assessment or consideration of the effect of the placebo effect in relation to the number of injections was considered within these analyses.

This review has provided a detailed evaluation of differences between injection regimens of IA-HA for knee pain in OA. Future studies should directly compare different injection regimens of IA-HA in head-to-head RCTs. Moreover, future studies should review alternative outcome measurements such as function, stiffness, and withdrawal rates due to the different number of IA-HA injections vs saline. Further studies should also aim to further compare the different HA products and review intrinsic efficacy and safety profiles of different products based on the number of injections and their molecular weight, structure, and production method. The identification of product-specific results would also allow for greater specificity in drafting clinical guidelines for use of HA in knee OA. Injection accuracy is also a factor that may contribute to the overall efficacy and safety profile of IA-HA treatment [55], which may warrant future investigation of how accuracy improvement using ultrasound-guided injection techniques may affect clinical outcomes in trials. Additionally, the use of pre-set criteria for response, such as the OMERACT-OARSI responder criteria, is an emerging outcome measurement tool that may provide a more appropriate assessment of individual patient outcomes rather than group mean responses [56]. However, the lack of consistent reporting of this outcome within the current literature precluded the ability to conduct any analysis of OMERACT-OARSI responders between injection regimens [56]. Future studies should consider analyzing measures of individual response according to present criteria for response when designing clinical trials in knee osteoarthritis, including those addressing the impact of IA-HA.

Other factors in addition to injection regimen may contribute to the efficacy and safety of IA-HA products, such as molecular weight and production process. Altman and colleagues [6] concluded that in the available literature, IA-HA products with a molecular weight ≥ 3000 kDa and those derived from biological fermentation relate to superior efficacy and safety [6]. The low molecular weight (LMW) IA-HA pooled effect size did not meet the minimum clinically important difference (MCID) threshold, demonstrating an insignificant clinical effect for pain relief. Due to the multiple variables that may contribute to the efficacy of IA-HA products, additional investigations comparing the different types of HA products are required to fully understand the efficacy differences of IA-HA products in knee OA. Guideline development groups and clinicians should consider the injection regimen for various types of IA-HA treatments in decision-making processes regarding the appropriate use of IA-HA treatment for knee OA.

## Conclusion

Overall, 2–4 and ≥5 injection regimens provided pain relief over IA-Saline. Intra-articular injections of HA used in a 2–4 injection treatment regimen provided the greatest benefit when compared to IA-Saline with respect to pain improvement in patients with knee OA, and were generally deemed safe with few to no treatment-related AEs reported across studies. Future research is needed to directly compare these treatment regimens, as well as further investigate the effects of other variables, such as product molecular weight, in the comparison of IA-HA injection treatment regimens.

## Additional files

Additional file 1: Literature Search Strategy. (DOCX 15 kb)

Additional file 2: Figure S1. Funnel plot analysis of studies investigating efficacy of IA-HA injections closest to 3-months. (DOCX 18 kb)

Additional file 3: Figure S2. Funnel plot analysis of studies investigating efficacy of IA-HA injections closest to 6-months. (DOCX 19 kb)

## Abbreviations

AAOS: American Academy of Orthopaedic Surgeons
ACR: American College of Rheumatology
AD: Avian-derived
AE: Adverse Events
AMSSM: American Medical Society for Sports Medicine
CI: Confidence interval
ESCEO: European Society for Clinical and Economic Aspects of Osteoarthritis
FDA: Food and Drug Administration
HWM: High molecular weight
IA-HA: Intra-articular hyaluronic acid
ISK: Index of Severity for Osteoarthritis for the Knee
KOOS: Knee Injury and Osteoarthritis Outcome Score
LMW: Low molecular weight
MCID: Minimum clinically important difference
MODEMS: Musculoskeletal Outcomes Data Evaluation and Management System
MW: Molecular weight
NSAID: Non-steroidal anti-inflammatory drug
OA: Osteoarthritis
OARSI: Osteoarthritis Research Society International
OMERACT: Outcome measures in rheumatology
PRISMA: Preferred Reporting Items for Systematic Reviews and Meta-Analyses
RCT: Randomized controlled trial
RR: Risk ratio
SAE: Serious adverse events
SMD: Standard mean difference
US: United States
VAS: Visual analog score
WOMAC: Western Ontario and McMaster Universities Arthritis Index
