# Supplementary material for: The efficacy of multiple versus single hyaluronic acid injections: a systematic review and meta-analysis
Source: BMC Musculoskelet Disord. 2017 Dec 21;18:542. doi: 10.1186/s12891-017-1897-2 (PMC5740709; doi:10.1186/s12891-017-1897-2)
Supplement: Supplementary file 1 — Literature Search Strategy. (DOCX 15 kb) [file 12891_2017_1897_MOESM1_ESM.docx]

Appendix 1: Literature Search Strategy

| **MEDLINE and EMBASE** | **PubMed** |
| --- | --- |
| 1. Hyaluronic acid[title] 2. Hylan[title] 3. Hyaluronan[title] 4. Viscosupplementation[title] 5. Osteoarthrit$.mp 6. Knee.mp 7. 1 or 2 or 3 or 4 8. 5 and 6 9. 7 and 8 10. Euflexxa.mp 11. Synvisc$.mp 12. Supartz.mp 13. Orthovisc.mp 14. Durolane.mp 15. Hyalgan.mp 16. 10 or 11 or 12 or 13 or 14 or 15 17. 9 or 16 18. Randomiz$ adj3 Control$ Trial 19. 17 and 19 | 1. Hyaluronic acid[title] 2. Hylan[title] 3. Hyaluronan[title] 4. Viscosupplementation[title] 5. Osteoarthrit$.mp 6. Knee.mp 7. 1 or 2 or 3 or 4 8. 5 and 6 9. 7 and 8 10. Euflexxa.mp 11. Synvisc$.mp 12. Supartz.mp 13. Orthovisc.mp 14. Durolane.mp 15. Hyalgan.mp 16. 10 or 11 or 12 or 13 or 14 or 15 17. 9 or 16 18. Randomiz$ adj3 Control$ Trial 19. 17 and 19 |
