# Supplementary figures and images for: The efficacy of multiple versus single hyaluronic acid injections: a systematic review and meta-analysis
Source: BMC Musculoskelet Disord. 2017 Dec 21;18:542. doi: 10.1186/s12891-017-1897-2 (PMC5740709; doi:10.1186/s12891-017-1897-2)

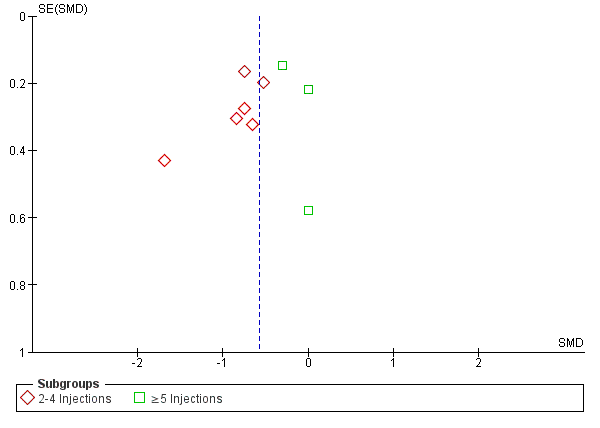

Supplement: Supplementary file 2 — Funnel plot analysis of studies investigating efficacy of IA-HA injections closest to 3-months. (DOCX 18 kb) [file 12891_2017_1897_MOESM2_ESM.docx]

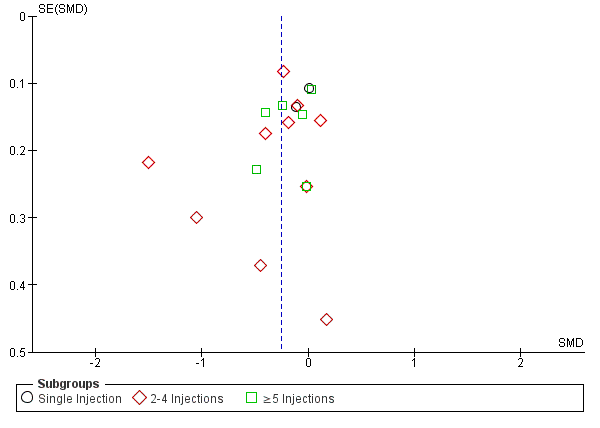

Supplement: Supplementary file 3 — Funnel plot analysis of studies investigating efficacy of IA-HA injections closest to 6-months. (DOCX 19 kb) [file 12891_2017_1897_MOESM3_ESM.docx]
